# Contributing factors to declining vaccination campaign quality and resurgence of polio cases in Quetta block, Pakistan: a five-year analysis (2020–2024)

**DOI:** 10.3389/fpubh.2026.1817720

**Published:** 2026-04-23

**Authors:** Ehsan Larik, Aftab Kakar, Kifayat Ullah, Tamkeen Ghafoor, Abdul Sami Khan, Gohar Ali Shahbaz, Abdul Raziq, Chukwuma Mbaeyi

**Affiliations:** 1 National Stop Transmission of Polio (NSTOP) Program, Islamabad, Pakistan; 2 Expanded Programme on Immunization, Balochistan, Pakistan; 3 Provincial Emergency Operation Centre (EOC), Polio Eradication Initiative, Balochistan, Pakistan; 4 Integral Global Health, Federal Directorate of Immunization, Islamabad, Pakistan; 5 Global Immunization Division, Centers for Disease Control and Prevention, Atlanta, GA, United States

**Keywords:** Pakistan, polio eradication, poliovirus, routine immunization, vaccination supplementary immunization activities, vaccine preventable diseases, vaccines

## Abstract

**Introduction:**

Implementation of supplementary immunization activities (SIAs) delivering oral poliovirus vaccine (OPV) to children under 5 years of age is a key strategy for interruption of poliovirus transmission. Quetta block (Quetta, Pishin, Chaman, and Killa Abdullah districts), which has the largest number of children under 5 years in Balochistan province, is a core reservoir for wild poliovirus type 1 (WPV1) in Pakistan. This study examined the quality of polio SIAs and trends in WPV1 cases in Quetta block during 2020–2024.

**Methods:**

We conducted descriptive analysis of administrative coverage, lot quality assurance sampling (LQAS) surveys and post-campaign monitoring (PCM) data for polio SIAs implemented during 2020–2024. Data on WPV1 cases and environmental isolates for the same period were obtained from the acute flaccid paralysis (AFP) and environmental surveillance databases.

**Results:**

Of the 33 SIAs planned in Quetta block during 2020–2024, only 24 (73%) were fully implemented. Due to the COVID-19 pandemic, all polio SIAs in Pakistan were paused in the first half of 2020 but resumed by July 2020. The quality of SIAs in Quetta block declined during the period under review, with the proportion of LQAS lots that achieved the threshold for acceptable performance (≥95%) reducing from 90% in 2020 to 70% in 2023. WPV1 cases re-emerged in 2024 in Quetta block after a three-year absence and a 28-month period without WPV1 isolations from environmental samples. The 14 WPV1 cases that were reported in Quetta block accounted for 19% of all 74 cases in Pakistan and 98% of environmental samples within the block tested positive for WPV1 in 2024.

**Conclusion:**

Quetta block in Balochistan province experienced a significant reduction in the quality of polio SIAs during 2020–2024, which likely contributed to a resurgence in WPV1 cases in 2024. Factors influencing the decline in SIA quality included COVID-19 disruptions of immunization activities, security incidents, community boycotts, and vaccine hesitancy. To enhance the prospects of poliovirus elimination in Balochistan, stronger government commitment and greater community engagement will help to improve vaccine access and acceptance.

## 1 Introduction

Implementation of supplementary immunization activities (SIAs) is one of the main strategies adopted by the Global Polio Eradication Initiative (GPEI) for the eradication of polio (1, 2). The oral poliovirus vaccine (OPV) is often the vaccine administered during SIAs, with the aim of reaching and vaccinating all eligible children under 5 years of age (3). Implementation of high-quality polio SIAs has contributed to eliminating the disease from all but two countries – Pakistan and Afghanistan. In Pakistan, several SIAs are implemented annually, nationally and sub-nationally, in concert with GPEI partner agencies supporting eradication efforts in the country (4). SIAs in Pakistan are implemented within 5 or 7 days depending on accessibility of the region of the country. In most areas, mobile vaccination teams visit all households within 3 days in line with a microplan developed to facilitate reaching and vaccinating all children under 5 years of age. Over the subsequent 2 days, the teams vaccinate any child that was unvaccinated during the previous days (3 + 2 strategy). In areas with difficult population access, socially or geographically, special mobile teams (SMTs) and community-based vaccination (CBV) teams have 5 days to visit all households and 2 additional days for catch-up vaccination of missed children (5 + 2 strategy). To facilitate SIA implementation, every union council (subdistrict) prepares a microplan for vaccination teams including detailed description of resources, expected target populations, and daily team movement plans by location.

Balochistan is the largest province of Pakistan by land mass, accounting for nearly 44% of the country’s total land area (5). Quetta block, consisting of Quetta, Pishin, Killa Abdullah and Chaman districts, is located in southern Balochistan and remains a core reservoir for poliovirus circulation with low immunization coverage (6). With an area of 14,559 square kilometers, the block shares an international border with the eastern provinces of Afghanistan and is the most populous region of Balochistan. Its geographic contiguity with the eastern provinces of Afghanistan is of significance as cross-border movements between both countries contribute to bidirectional transmission of poliovirus to and from Balochistan (4). During 2020–2024, its total population was over 4.25 million persons, with approximately 810,000 children under 5 years of age distributed among about half a million households, all of which need to be visited during every polio vaccination campaign (7). The quality of health infrastructure in Quetta block is substandard, and the rugged landscape and scattered population create significant barriers to healthcare delivery including vaccination services. Socio-economic issues such as high poverty rates, low literacy levels, and ongoing political unrest further complicate vaccination efforts (8). Due to these factors, high numbers of eligible children are unvaccinated through the national essential immunization program. Routine vaccination coverage in Balochistan lags considerably behind other provinces in Pakistan and was estimated to be as low as 38% in 2022 (9). As a result, the province is at high risk of persistent poliovirus circulation and is even more reliant on SIAs than others in the country to compensate for substantial deficiencies in routine vaccination coverage. This has heightened the risk of continued poliovirus circulation in the Quetta block area of the province.

Implementation of polio SIAs in Quetta block has been marked by significant challenges for a variety of reasons, including security, socio-cultural, and infrastructural reasons, all of which were exacerbated during the COVID-19 pandemic. Furthermore, despite being one of the core polio reservoirs in Pakistan, Quetta block adopted the 3 + 2 campaign strategy instead of the 5 + 2 strategy, which limited the amount of time available for vaccination teams to revisit and vaccinate missed children, potentially increasing the workload of teams to the detriment of vaccination campaign quality (9). Continued poliovirus circulation in Pakistan is partly due to a failure to consistently conduct high-quality SIAs in such priority areas of the country for the polio program (10). Available research demonstrates the impact of SIAs in improving population immunity in areas with weak or non-existent routine immunization systems such as Quetta block (11). However, the same factors impeding the delivery of routine immunization services often impair the quality of polio SIAs in Quetta block. Factors such as lack of community engagement and parental misconceptions about vaccines pose significant barriers to achieving high coverage (12). Logistical challenges for cold chain management of vaccines and transportation difficulties are also critical obstacles to implementing high-quality SIAs in the region.

Against this backdrop, a resurgence of WPV type 1 (WPV1) cases occurred in Quetta block in 2024 after a relatively quiescent three-year period during 2021–2024 (13). Beginning in the second half of 2020, a gradual attenuation in the number of reported WPV1 cases was observed across Pakistan, coincident with the onset of the COVID-19 pandemic and attendant control measures put in place by the government. To better understand the reasons for the resurgence in WPV1 cases in 2024, we conducted a study assessing the number, scope and quality of polio SIAs in Quetta block during 2020–2024 and examined potential reasons for suboptimal polio campaign quality.

## 2 Materials and methods

### 2.1 Study design

We conducted a retrospective descriptive study of polio vaccination campaigns conducted during January 2020 to December 2024 to identify factors contributing to the resurgence of WPV1 circulation in Quetta block of Balochistan.

### 2.2 Study area and population

The study was conducted in Quetta block of Balochistan province, comprising four geographically contiguous districts, namely Quetta, Chaman, Killa Abdullah and Pishin, considered of high priority to the polio program (14).

### 2.3 Data sources

Data on polio SIAs conducted during the study period were extracted from the polio dashboard managed by the National Emergency Operations Center (NEOC). SIA databases included administrative coverage data, polio campaign market surveys, postcampaign monitoring (PCM) assessments, and lot quality assurance sampling (LQAS) survey data. To ensure data accuracy and completeness, the data were crosschecked for duplication and inconsistencies and missing values were removed. Poliovirus cases were identified through laboratory testing of stool samples collected from acute flaccid paralysis (AFP) cases, encompassing all causes of AFP not limited to suspected polio cases. Additionally, poliovirus circulation within the community was also detected through the collection of sewage samples from designated environmental surveillance (ES) sites. AFP and environmental surveillance data were obtained from the Information for Action (IFA) database managed by the national polio program.

### 2.4 Data analysis

A secondary data analysis was conducted using Microsoft Excel. We enumerated polio SIAs conducted in Quetta block during the study period. Descriptive analysis of administrative data was conducted to calculate the number of children under 5 years of age vaccinated during each campaign and percentages of district-wise and annual SIA coverage during the period under review. In addition, trends in numbers and proportions of still missed children, and results of LQAS surveys (Table 1) and PCM assessments, were used to gain a more accurate picture of the quality of polio SIAs during the study period. Still missed children are those children, recorded as unvaccinated at the time of the initial visit, who remain unvaccinated at the completion of an SIA after efforts to revisit their homes and vaccinate them. Among these children, still refusals are those children recorded as refusals by vaccination teams that are not vaccinated during catch-up campaign days while still leftover children are those in targeted areas that were not covered due to security reasons, boycotts, or accessibility issues.

**Table 1:** Lot quality assurance sampling (LQAS) survey performance by district in Quetta Block, Balochistan, Pakistan, 2020–2024.

District	Year	% Pass LQAS lots	Number of LQAS lots pass	Total LQAS lots conducted
Chaman	2020	88%	21	24
	2021	86%	37	43
	2022	64%	27	42
	2023	50%	8	16
	2024	70%	21	30
Kabdulah	2020	94%	17	18
	2021	80%	36	45
	2022	58%	29	50
	2023	33%	10	30
	2024	46%	22	48
Pishin	2020	98%	53	54
	2021	98%	80	82
	2022	83%	50	60
	2023	85%	34	40
	2024	95%	70	74
Quetta	2020	82%	41	50
	2021	94%	77	82
	2022	80%	72	90
	2023	82%	56	68
	2024	91%	98	108
Quetta block	2020	90%	132	146
	2021	91%	230	252
	2022	74%	178	242
	2023	70%	108	154
	2024	81%	211	260

To assess SIA quality and coverage, LQAS surveys and PCM assessments are conducted after each campaign in Pakistan. LQAS surveys involve randomly selecting at least six lots (clusters) at the union council level and then sampling 10 households within each cluster to check for finger-marking (proof of vaccination) of a randomly selected child in the household to confirm that the child was vaccinated during the SIA. LQAS lots are classified as passing or failing based on whether the observed number of vaccinated children exceeds a predefined threshold for determining acceptable performance (≥95%). PCM assessments provide community-level estimates of vaccinated children under 5 years of age (15). During LQAS and PCM surveys, interviewers also record reasons for missing vaccination among children found to be unvaccinated, including operational reasons such as absence of the child at the time the vaccination team visited the household or failure of the vaccination team to visit the household. Finally, we analyzed trends in WPV1 cases and positive environmental surveillance isolates and triangulated these results with data on polio SIA quality.

### 2.5 Ethical considerations

Written permission for the study was obtained from the coordinator of the provincial polio emergency operations center (PEOC) in Balochistan. Guidelines for use of secondary data were followed. All personal identifiers were removed from data prior to analysis to ensure confidentiality. Ethical standards in reporting and interpreting findings were strictly followed to ensure transparency and accuracy. This activity was reviewed by CDC, deemed not research, and was conducted in line with applicable federal law and CDC policy. Because this study was conducted as part of a program evaluation activity, it was not deemed to be human subjects’ research and informed consent was not required or obtained.

## 3 Results

### 3.1 Supplementary immunization activities

**Figure 1:**
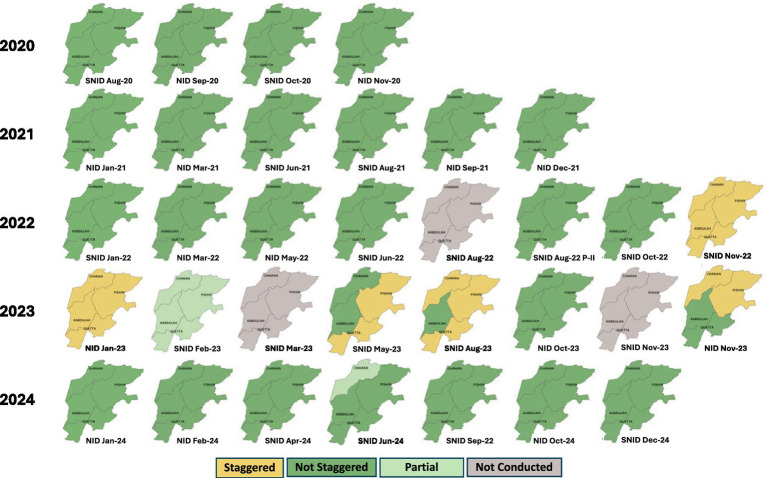
Supplementary immunization activities (SIAs) implemented in Quetta Block, Balochistan Province, Pakistan, 2020–2024. *Staggered: Polio campaigns arranged at different times and campaigns in the district completed in more than 5–7 days.

Between 2020 and 2024, 33 polio SIAs were planned for Quetta block. Of these, 24 (73%) were implemented as scheduled in a single phase, 5 (15%) were implemented using a staggered approach, with different areas conducting activities at different times instead of simultaneously due to security challenges, 1 (3%) was partially completed, and 3 (9%) were canceled (Figure 1). At the district level, in Chaman, 4 SIAs were staggered and 3 canceled; in Killa Abdullah, 2 were staggered and 2 canceled; and in Quetta and Pishin combined, 5 were staggered and 2 canceled. The higher number of canceled SIAs at the district level (*n* = 7) compared to the block total (*n* = 3) reflects overlap, as the same canceled SIA affected multiple districts within the block.

**Figure 2:**
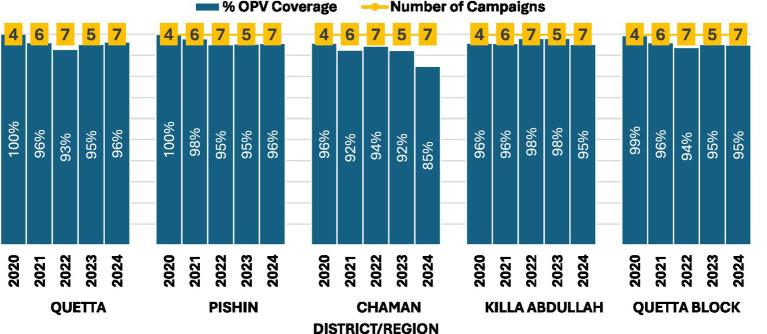
District-wise average administrative coverage during polio supplementary immunization activities (SIAs) in Quetta Block, Balochistan Province, Pakistan, 2020–2024.

Of the 33 polio SIAs planned for Quetta block during the study period, 14 (42%) were going to be part of national immunization days (NIDs), 16 (48%) were supposed to be part of subnational immunization days (SNIDs), and the remaining were to be local vaccination activities in response to WPV1 cases or environmental surveillance isolates in the area. Because of the pause in vaccination activities related to the COVID pandemic, campaigns did not take place between April and June 2020 resulting in about 2–3 missed campaigns during that year. Sit-ins and boycotts compromised the quality of 3 polio SIAs in Chaman district between November 2022 and December 2024. Furthermore, one SIA scheduled for August 2022 did not take place due to flooding and another scheduled for June 2024 was canceled due to attacks on polio workers. Based on administrative data, average campaign coverage for all SIAs conducted during the period under review in Quetta block was ≥ 95% during 2021, 2023 and 2024 but was 94% during 2022. At the district level, average SIA coverage decreased from 96% in 2020 to 85% in 2024 in Chaman, whereas in other districts coverage remained above 95% in nearly all years (Figure 2).

The proportion of still missed children during SIAs conducted in Quetta block was 10% in 2020 and decreased to 5% in 2024, but it did not meet the national benchmark of <0.75%. The reasons for still missed children commonly included that the child was not available at the time of the vaccinators’ visit, refusals to vaccinate and children living in leftover areas not covered by vaccination teams for security or other accessibility reasons (Table 2). The district of Chaman had the highest number of still missed children during the study period and the number increased from 37,903 in 2020 to 109,489 in 2024. Boycotts in the district resulted in 66,826 (76% of target) children being unreached during the June 2024 SNIDs. In Killa Abdullah district, 15,957 (23.5% of target) eligible children were unreached due to security issues during polio SIAs in April 2024.

**Table 2:** Proportions of still missed children during polio supplementary immunization activities (SIAs) in Quetta Block, Balochistan Province, Pakistan, 2020–2024.

Districts	Year	Target <5Y children	% Still NA	% Still refusals	% Still leftover	% Still missed
Chaman	2020	284,690	9.6%	3.7%	0.0%	13.3%
	2021	463,929	6.2%	2.3%	0.0%	8.5%
	2022	593,405	6.0%	1.5%	0.0%	7.5%
	2023	427,741	5.6%	3.1%	1.8%	10.5%
	2024	615,104	4.8%	1.8%	11.2%	17.8%
Killa Abdullah	2020	262,164	5.0%	2.5%	0.0%	7.5%
	2021	398,733	3.4%	1.3%	0.0%	4.7%
	2022	448,137	3.5%	0.6%	0.0%	4.1%
	2023	329,257	3.9%	0.7%	0.0%	4.6%
	2024	476,252	3.2%	0.4%	3.4%	7.0%
Pishin	2020	541,979	4.9%	0.8%	0.0%	5.7%
	2021	857,859	3.3%	0.4%	0.0%	3.8%
	2022	1,057,062	3.6%	0.4%	0.0%	3.9%
	2023	758,035	3.6%	0.3%	0.0%	3.9%
	2024	1,052,870	2.8%	0.3%	0.0%	3.1%
Quetta	2020	1,887,246	8.6%	2.3%	0.0%	10.9%
	2021	3,040,719	7.1%	1.3%	0.0%	8.4%
	2022	3,777,761	5.3%	0.5%	0.0%	5.8%
	2023	2,583,330	3.7%	0.2%	0.0%	4.0%
	2024	3,586,562	2.9%	0.2%	0.0%	3.1%

*NA: Not available (eligible child was not around at the house during visit of vaccination team). * < 5Y: Children <5 years of age eligible for polio vaccination. *Still NA: Recorded not available child by team who was not vaccinated during catch-up campaign days. *Still Refusal: Recorded refusal child by vaccination team not vaccinated during catch-up campaign days. *Still Leftover: Targeted area that was not covered by team due to security reasons, boycott, accessibility issues.

**Figure 3:**
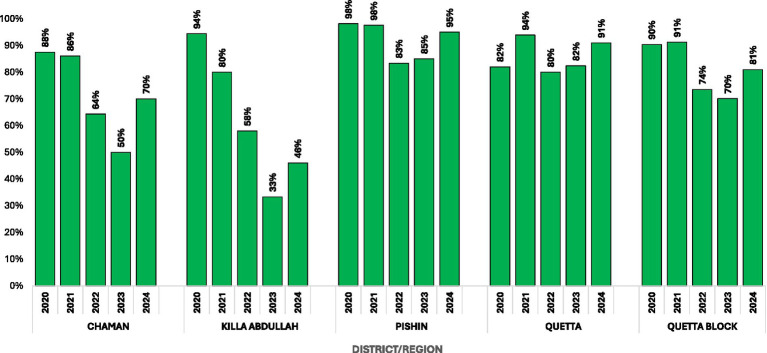
Results by district of lot quality assurance sampling (LQAS) surveys conducted after polio supplementary immunization activities (SIAs) in Quetta Block, Balochistan, Pakistan, 2020–2024.

LQAS surveys, which provide the most rigorous metric for assessing the quality of polio SIAs, showed that the percentage of lots (i.e., lower administrative units) that achieved the recommended pass mark of ≥95% reduced from 90% in 2020 to 70% in 2023 in Quetta block. At the district level, Killa Abdullah showed steep declines in LQAS results over the study period, with 94% of lots achieving a pass mark in 2020 compared with 33% in 2023. Chaman had 86–88% of lots passing in 2020–2021 compared with 50% in 2023 and 70% in 2024. In Pishin and Quetta the decline in lots achieving a pass was less significant (declines of 13% for Pishin and 12% for Quetta comparing pass rates in 2021 to 2023; Figure 3).

**Figure 4:**
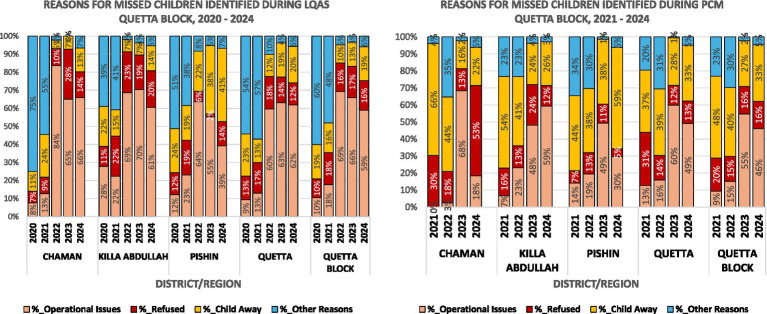
Reasons for children missing vaccination during polio supplementary immunization activities (SIAs) based on data from post campaign monitoring (PCM) and lot quality assurance sampling (LQAS) surveys conducted in Quetta Block, Balochistan, Pakistan.

In parallel with the worsening of LQAS survey results during the study period, we observed an increase in the proportion of children missed because of operational reasons, from 10% in 2020 to 59% in 2024 in all of Quetta block (Figure 4). Similar trends were observed in all four districts, although the change was more marked in Chaman where the percentage of children missed due to operational reasons increased from 8% in 2020 to 84% in 2022 and was 65–66% during 2023–2024. Operational reasons for missing children included a failure of vaccination teams to visit houses with eligible children, absence or illness of the child at the time of the team’s visit, and inadequate microplans that did not include houses with eligible children.

### 3.2 WPV1 cases, AFP and environmental surveillance

**Figure 5:**
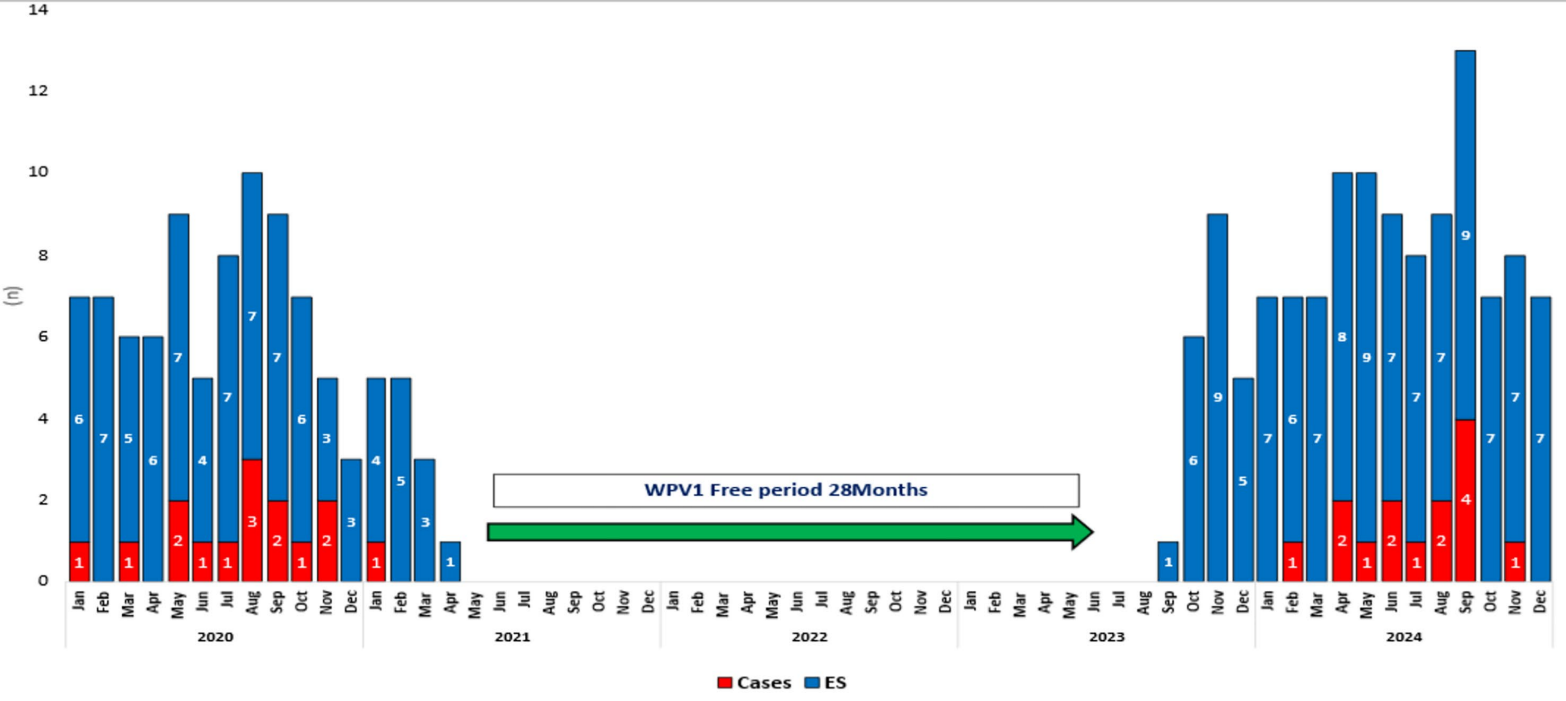
Poliovirus epidemiology showing wild poliovirus type 1 (WPV1) cases and environmental surveillance (ES) isolates, Quetta Block, Balochistan, Pakistan, 2020–2024.

During 2020, 84 WPV1 cases were reported in Pakistan, but only a single WPV1 case was reported in all of Pakistan in 2021 (1). The year 2021 marked the beginning of a period of relative quiescence in poliovirus circulation in Quetta block, as was the case in many parts of Pakistan, with no new WPV1 case being reported during February 2021 to January 2024 (Figure 5). Concomitantly, all the 439 ES samples collected during a 28-month period from May 2021 to August 2023 were negative for WPV1. In September 2023, WPV1 was detected again in ES samples, preceding a resurgence in WPV1 cases and ES isolates in 2024. During 2024, a total of 14 WPV1 cases were reported from Quetta block, including 7 cases from Killa Abdullah, 2 from Chaman, 2 from Pishin and 3 from Quetta. This represented 19% of the 74 WPV1 cases reported in Pakistan that year. The number of ES samples positive for WPV1 was 88/90 (98%) in Quetta block in 2024. Among the WPV1 cases, 79% had not received any polio vaccine through routine immunization and 36% had received less than 3 doses of OPV through SIAs. Key AFP surveillance indicators in the province of Balochistan were above recommended benchmarks during 2020–2024 (14).

## 4 Discussion

Quetta block, comprising Chaman, Killa Abdullah, Pishin, and Quetta districts, has historically been a core reservoir for poliovirus circulation in Pakistan. In mid-2024, the area experienced a resurgence of wild poliovirus transmission. With the largest population of children under 5 years in Balochistan, the block accounted for nearly one-fifth of the WPV1 cases reported nationally in 2024. This resurgence was likely driven by a historic decline in the quality of SIAs. LQAS surveys, which provide one of the most rigorous metrics of vaccination campaign quality, showed a steep decline in the quality of polio SIAs during the preceding 3 years, with the proportion of lots achieving a pass mark in Quetta block declining from 90% in 2020 to 70% in 2023. Declines in LQAS performance were even steeper in the security-compromised Killa Abdullah district, where the number of lots achieving a pass mark declined dramatically from 94% in 2020 to just 33% in 2023. Concomitantly, the percentage of children missed due to operational reasons during polio SIAs in Quetta block rose from 10% in 2020 to 59% in 2024.

The decline in the quality of vaccination campaigns was brought on by a number of factors including staggered implementation and cancelations of campaigns, security challenges, community boycotts, flooding, large-scale population movements due to repatriation of Afghan nationals, socio-cultural events (national games), and a national census. Studies by Ullah et al. and Buffarini R et al. have shed light on the contribution of these factors to decreased vaccination (16, 17). While the COVID-19 pandemic caused a national disruption of vaccination activities, leading to suspension of all polio SIAs in March 2020 (18), vaccination activities resumed by July 2020 and polio SIAs were implemented in line with the schedule by 2021. Although fear of COVID-19 influenced the attitude of health workers and the community toward participation in vaccination activities (19, 20), it appears from available data that it may not have been a major driver of the decline in vaccination coverage during the succeeding years. If anything at all, Pakistan’s healthcare workforce demonstrated its resilience through a robust response to the pandemic and prompt resumption of polio SIAs and other immunization activities (21).

Paradoxically, the advent of the COVID-19 pandemic ushered in a period of quiescence in poliovirus circulation in Quetta block. No WPV1 case was identified in the block for a three-year period spanning from February 2021 to January 2024 despite maintenance of adequate AFP surveillance as demonstrated by performance indicators. Even the more sensitive environmental sewage sampling system did not pick up any WPV1 isolates in the block for a 28-month period during May 2021 to August 2023. Apparently, the quiescence in poliovirus transmission in Quetta block might have also contributed to a false sense of security about the absence of poliovirus circulation in the area, leading to a decline in the quality of polio SIAs exacerbated by security-related and socio-cultural factors. This would have led to a build-up of polio susceptible children in the block, allowing for a resurgence of virus transmission regardless of whether this occurred through recrudescence of local transmission or importation of the virus from other infected locations.

Staggered implementation of vaccination campaigns in areas with compromised access led to inconsistencies in coverage. Implementing SIAs sequentially across districts, i.e., one after the other, instead of simultaneously, compromised uniformity in planning, logistics, and supervision, leading to inconsistencies in overall coverage. Furthermore, the switch from the 5 + 2 days to 3 + 2 days campaign strategy resulted in a number of operational failures, including the inability of vaccination teams to visit houses with missed children. This situation was further complicated by community boycotts and ongoing security concerns in the block. Sporadic and targeted attacks on polio workers necessitated security deployments during polio SIAs in the block (22). The repatriation of Afghan refugees, scheduling of national games, and the national census of 2022 created an additional burden on efforts to enumerate and access all targeted children during campaigns and in ensuring security for vaccination teams during polio SIAs. These factors collectively hampered the successful implementation of polio vaccination activities in the region (23).

The disruptions of polio vaccination campaigns in Quetta block during 2020–2024 spotlight the challenges faced in countries and regions confronted with similar obstacles and experiencing uninterrupted poliovirus transmission or recurrent outbreaks. For instance, Afghanistan, the other polio-endemic country, and Nigeria, currently experiencing polio outbreaks, have persistent low immunization coverage in certain regions due to chronic insecurity, community protests, vaccine hesitancy and pandemic-related issues (24, 25). To address such issues, in the past countries like India and Ethiopia conducted high-quality campaigns based on meticulous microplanning to achieve poliovirus elimination (26). Applying the strategies adopted by these countries within the broader Global Polio Eradication Initiative framework could help to reach and vaccinate vulnerable children, reducing the number of polio cases and outbreaks (27). Flexibility in campaign schedules is also essential as they allow for continuation of vaccination activities under certain extenuating circumstances. Flexible schedules allowed for immunization activities in the Democratic Republic of Congo (DRC) and Somalia to continue despite COVID-19 pandemic disruptions and could be used in Pakistan to reduce coverage gaps and compensate for campaign cancelations due to security concerns (28, 29).

Another factor that played a key role in the deterioration of polio SIA quality in Balochistan was community boycotts. Sit-in protests in Chaman from November 2023 to July 2024 led to campaign boycotts that resulted in a deterioration in overall SIA campaign quality in the district and Quetta block as a whole. When similar challenges were encountered in conflict-affected regions of Somalia, the country’s polio program responded promptly and worked with local community leaders and international organizations to overcome community hesitancy and sustain support for high-quality vaccination campaigns (30, 31). Conversely, the response to local disruptions in Balochistan was slow and lacked significant government commitment, which may have led to missed opportunities to reach and vaccinate eligible children during the period under review. A more flexible and resilient campaign strategy, along with stronger community and government ownership and trust-building, may improve the quality of future immunization activities in Quetta block.

The resurgence of poliovirus circulation in Quetta block in 2024, while reflective of broader patterns of poliovirus transmission in Pakistan, demonstrates the unique obstacles to achieving adequate vaccination coverage in resource-constrained and security-compromised settings. Community hesitancy, natural disasters, and political instability contributed to the decline in immunization efforts, hence allowing the virus to persist and re-emerge. Although conflict and public health crises also affected polio and routine immunization activities in other countries (29), Quetta block’s situation was especially challenging due to its geographic, managerial, and political barriers, which slowed polio outbreak responses despite early warnings from environmental surveillance. Studies from Khyber Pakhtunkhwa province also highlight this issue where, despite early detection, delays in responding to initial poliovirus detections reduced the chances for controlling the spread of the virus within the province. Application of best practices from other countries on community engagement and flexible immunization strategies can guide efforts to improve responses to outbreaks and ameliorate the problem of persistently missed children and refusals during polio SIAs (32).

### 4.1 Limitations and public health implications

Our study findings are specific to Quetta block and may not be generalizable to other districts of Balochistan and provinces in Pakistan. Our findings are also dependent on the quality of the secondary data sources, which may be affected by data discrepancies especially in estimating population denominators. However, overall trends in our data, particularly as indicated by LQAS surveys, suggest that the decline in polio SIA coverage in Quetta block was unlikely to be artefactual. Our study demonstrates how socio-cultural and geo-political factors within a resource-limited setting can influence vaccination coverage and the dynamics of poliovirus transmission. These findings will contribute to a broader understanding of effective strategies for improving child health outcomes and informing future public health interventions.

## 5 Conclusion

The study highlights the complex interplay of factors affecting polio SIA coverage in Quetta block, leading to a resurgence of wild poliovirus cases. A number of extraneous factors contributed to compromised SIA quality in Quetta block during 2020–2024 that likely resulted in a surge in poliovirus circulation. While there has been some progress in addressing these factors, particularly through improved SIA planning and surveillance efforts, the pace of progress has been slow and persistent challenges remain. Addressing these challenges requires a multifaceted approach involving coordination among all provinces in Pakistan on polio eradication activities, government commitment, international support, community engagement, and innovative solutions to close gaps in vaccination coverage. Strengthening health system resilience through better infrastructure, emergency contingency plans, and addressing sanitation and malnutrition through integrated service delivery will complement polio eradication efforts. By understanding and addressing the specific barriers to polio vaccination in Balochistan, the goal of poliovirus elimination in the region can be achieved, contributing to the broader global effort to eradicate polio.

## Data Availability

The data analyzed in this study is subject to the following licenses/restrictions: the original contributions presented in the study are included in the article. Further inquiries can be directed to the corresponding author. Requests to access these datasets should be directed to Chukwuma Mbaeyi, cmbaeyi@cdc.gov.
